# The role of midkine in health and disease

**DOI:** 10.3389/fimmu.2023.1310094

**Published:** 2023-11-30

**Authors:** Emely Elisa Neumaier, Veit Rothhammer, Mathias Linnerbauer

**Affiliations:** Department of Neurology, University Hospital Erlangen, Friedrich-Alexander University Erlangen-Nürnberg, Erlangen, Germany

**Keywords:** midkine, CNS, inflammation, malignancy, injury

## Abstract

Midkine (MDK) is a neurotrophic growth factor highly expressed during embryogenesis with important functions related to growth, proliferation, survival, migration, angiogenesis, reproduction, and repair. Recent research has indicated that MDK functions as a key player in autoimmune disorders of the central nervous system (CNS), such as Multiple Sclerosis (MS) and is a promising therapeutic target for the treatment of brain tumors, acute injuries, and other CNS disorders. This review summarizes the modes of action and immunological functions of MDK both in the peripheral immune compartment and in the CNS, particularly in the context of traumatic brain injury, brain tumors, neuroinflammation, and neurodegeneration. Moreover, we discuss the role of MDK as a central mediator of neuro-immune crosstalk, focusing on the interactions between CNS-infiltrating and -resident cells such as astrocytes, microglia, and oligodendrocytes. Finally, we highlight the therapeutic potential of MDK and discuss potential therapeutic approaches for the treatment of neurological disorders.

## Introduction

Growth factors are essential for the development and functioning of the central nervous system (CNS). As soluble molecules, they play vital roles in cell-to-cell communication and regulate a multitude of functions, including cell proliferation and differentiation. One of these growth factors is the heparin-binding growth factor midkine (MDK). MDK, together with the structurally related growth factor pleiotrophin (PTN), belongs to the family of neurite promoting growth factors and has originally been identified in embryonal carcinoma (EC) cells in 1988 (1).

MDK is expressed in a small number of embryonic tissues, including the CNS. The expression pattern of MDK during mouse gestation indicates that the growth factor is required for the generation of epithelial tissue, remodeling of the mesoderm (2), and neurogenesis (3). Early studies by Kadomatsu et al. (2) describe an upregulation of MDK during midgestation in mouse embryos, while its expression in adult mice has initially only been described in the kidney. The polypeptide MDK with a molecular weight of about 13 kDa (4) consists of a N-terminal domain, held together by three disulfide bridges and a C-terminal domain, stabilized by two disulfide bridges (5). Notably, early studies have suggested that the neurite outgrowth promoting functions of MDK are highly dependent on both the C-terminally located heparin-binding domain and the sulfide bonds (6–8).

**Figure 1:**
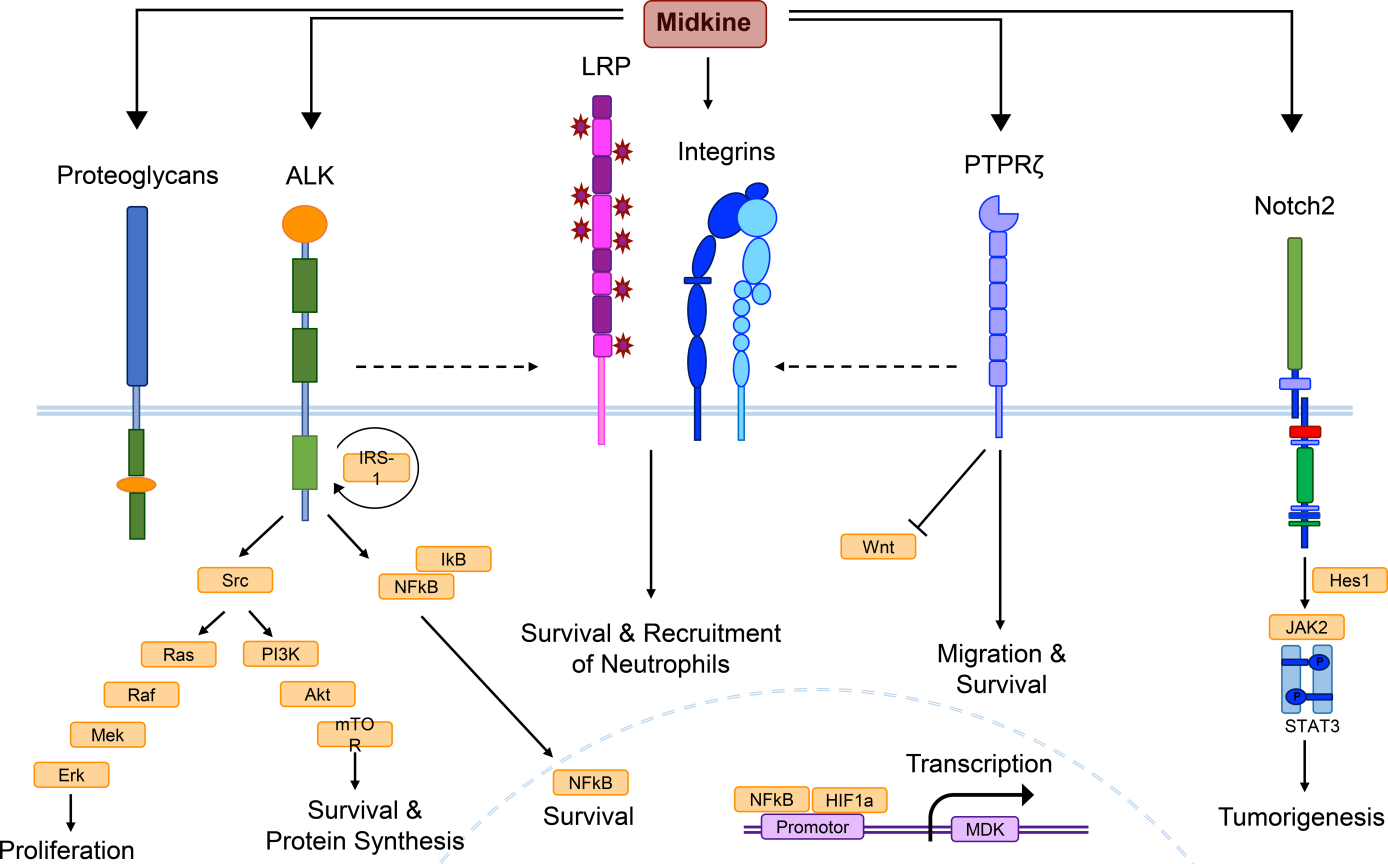
MDK receptor candidates and signaling pathways. Midkine (MDK) is a multifunctional molecule, whose effects are probably regulated via different receptor-ligand interactions, as well as complex formation of receptor candidates, and cross-talk between the receptors. Low density lipoprotein receptor-related proteins (LRPs) and integrins are thought to build the core of the MDK receptor complex, while other candidates such as the anaplastic lymphoma kinase (ALK) or the protein tyrosine phosphatase ζ (PTPζ) might be recruited. This figure combines signaling pathways discovered in different cell types under several pathological conditions and does not show the determined signaling of MDK in a specific cell type. Binding of MDK to the ALK receptor induces the phosphorylation of the insulin receptor substrate-1 (IRS-1) and its interaction with the ALK receptor, followed by the activation of several signaling pathways. Src kinase phosphorylation results in mitogen-activated protein (MAP)-kinase signaling, which includes a phosphorylation cascade of the proteins Ras, Raf, Mek, and Erk, supporting cell proliferation. Another downstream effect of Src is the phosphoinositide (PI)-3-kinase signaling, including Akt and mTOR activation, promoting survival and protein synthesis. MDK/ALK signaling also induces the expression of nuclear factor kappa light-chain enhancer of activated B cells (NF-κB), a growth factor inhibited by IκB proteins until it reaches the nucleus, where it stimulates cell survival. The core complex of LRP and integrins contributes to cellular survival and the recruitment of neutrophils, while MDK binding to PTPζ additionally promotes survival, as well as cell migration and negatively regulates Wnt signaling. Neurogenic locus notch homolog protein 2 (Notch2) activation mediates the interaction between Hes1 and the Janus kinase 2 (Jak2)/STAT3 complex, inducing tumorigenesis. The promotor of the MDK gene entails binding sites for NF-κB and hypoxia-inducible factor-1α (HIF-1α).

Recent research has indicated that MDK is highly upregulated in response to various pathological conditions, both in the CNS and the periphery (9, 10) (**Figure 1**, **Table 1**), and can be exploited as a biomarker and therapeutic target (35, 36), highlighting the pivotal role of the growth factor in the context of disease. In this review, we will summarize the involvement and function of MDK in the context of peripheral disorders and CNS pathologies, including brain injuries, brain tumors, as well as neuroinflammatory and neurodegenerative diseases. Furthermore, we will review existing therapeutic strategies targeting MDK in neoplastic diseases and discuss the therapeutic value of MDK for the treatment of CNS disorders.

**Table 1:** The major roles of MDK in pathological conditions.

Disease	Effects	Models	References
Alzheimer’s disease	Inhibits Aβ fibril formation and Aβ-induced cytotoxicity	*in vitro*	(11, 12)
Cardiac ischemia-reperfusion injury	Prevents myocardial apoptosis	*in vivo*	(13)
Cerebral infarct	Reparative neurotrophic functions during early phase	*in vivo*	(14)
Gastric cancer	Confers chemoresistance	*in vitro*	(15–17)
Glioblastoma	Induces stem-like properties of glioma initiating cells; induces cannabinoid resistance; modulates immunosuppressive tumor microenvironment	*in vitro* *in vivo*	(17–20)
Inflammatory breast cancer	Recruits monocytes	*in vitro*	(21)
Leukemia	Promotes B cell survival	*in vivo*	(22)
Melanoma	Modulates tumor microenvironment towards tolerogenic and immune-resistant states	*in vitro* *in vivo*	(23)
Multiple Sclerosis	Suppresses expansion of T_reg_ cells, deteriorating disease course	*in vivo*	(24, 25)
Neuroblastoma	Promotes tumorigenesis	*in vitro* *in vivo*	(26, 27)
Neuromyelitis optica	Correlates with IL-23 levels	*in vivo*	(28)
Pancreatic cancer	Contributes to chemoresistance, proliferation and migration of cancer cells	*in vitro*	(15, 16, 26, 27)
Renal ischemia-reperfusion injury	Promotes migration of neutrophils and macrophages	*in vivo*	(29)
Rheumatoid arthritis	Leads to activation and migration of neutrophils and inflammatory leukocytes; induces release of pro-inflammatory cytokines	*in vivo*	(18–20, 30, 31)
Systemic lupus erythematosus	Correlates with increased IL-17 levels	*in vivo*	(32)
Transient forebrain injury	Promotes tissue repair	*in vivo*	(33)
Traumatic brain injury	Polarizes microglia towards an anti-inflammatory state; recruits neutrophils and macrophages; increases apoptotic neurons around lesions; potentiates secondary injury	*in vivo*	(34)

## Cellular sources of MDK

In the periphery, numerous cell types have been identified to produce MDK under basal and pathological conditions (9). In addition to monocytes, macrophages, and monocyte-derived dendritic cells (mDCs), also non-hematopoietic cell types such as endothelial cells are capable of producing MDK (4).

Within the CNS MDK is mainly expressed during development until midgestation, while its mRNA levels decrease in postnatal life (4, 9). In mice MDK is mainly expressed by oligodendrocyte precursor cells (OPCs), followed by fetal astrocytes, neurons, and newly formed oligodendrocytes, while in humans fetal astrocytes represent the major source of MDK in the CNS (9). Moreover, *in vitro* studies show MDK expression by cultured neurons and activated astrocytes, but not microglia (37).

## Inducers of MDK expression

In monocytes, polymorphonuclear neutrophils (PMNs), and endothelial cells, MDK expression is induced during hypoxia (38). Binding of hypoxia-inducible factor-1α (HIF-1α) to hypoxia response elements (HREs) in the *MDK* promotor activates expression of the gene (**Figure 1**), while MDK in turn increases HIF-1α expression in a positive feedback loop (39). In addition to hypoxia, MDK expression is driven by the master regulator of pro-inflammatory pathways, nuclear factor kappa light-chain enhancer of activated B cells (NF-κB) (**Figure 1**), which can be activated by reactive oxygen species (ROS), pro-inflammatory cytokines such as tumor necrosis factor α (TNFα), interleukin (IL)-1β, as well as bacterial components, such as lipopolysaccharide (LPS) (9), among others. Together, both its regulation by hypoxia and pro-inflammatory NF-κB signaling indicate the relevance of MDK signaling in response to inflammatory stimuli.

While numerous studies have shed light on the role of MDK in non-CNS diseases, the regulation of MDK in CNS pathologies is less defined (**Table 1**). During development of experimental autoimmune encephalomyelitis (EAE), a preclinical animal model of Multiple Sclerosis (MS), T helper (T_H_) cells have been identified as MDK producer cells (24). However, in glial cells it can be speculated that similar processes drive the expression of MDK. In these lines, hypoxic conditions following ischemia, or the presence of pro-inflammatory cytokines such as TNFα and IL-1β under neuroinflammatory conditions (40, 41) may induce NF-κB-dependent upregulation of MDK by astrocytes, microglia, or oligodendrocytes. Whether this upregulation in fact occurs and whether it is part of a protective or inflammatory activation state must be addressed by future studies.

## MDK receptors and signaling

So far, several plasma membrane molecules have been identified as MDK receptors, including integrins, proteoglycans, neurogenic locus notch homolog protein 2 (Notch2) (42), ALK (43), low-density lipoprotein receptor-related protein (LRP) (44), and protein tyrosine phosphatase ζ (PTPζ) (45) (**Figure 1**). Integrins with MDK-binding properties include the heterodimers α_6_β_1_ and α_4_β_1_, while the family of MDK-binding proteoglycans can be subdivided into syndecans (46), glypican-2 (47), PG-M/versican (48), and neuroglycan C (49).

Instead of binding to a single one of these receptors, MDK exerts its multifaceted functions through binding to a multimolecular receptor complex (**Figure 1**), with PTPζ as the most established component (4, 50). The formation of the receptor complex, the arrangement of MDK-binding molecules, and the crosstalk between receptor subunits coordinate the signal transduction in response to MDK binding via several signaling pathways, depending on the cellular context, thereby facilitating the diverse functions of the growth factor (51) (**Figure 1**).

### Protein tyrosine phosphatase ζ

The signaling cascade elicited through binding of MDK to the receptor component PTPζ has been associated to various functions and cell types. Binding of macrophage migration inhibitory factor (MIF) to its receptor CD74 on mature and malignant B cells leads to an increased expression of MDK, which in turn increases B cell survival by autocrine MDK-signaling through PTPζ (22). Furthermore, the receptor PTPζ mediates MDK signals that suppress osteoblast proliferation via negative regulation of Wnt signaling (**Figure 1**) by dephosphorylation of β-catenin (52). However, it is not known whether MDK itself is able to induce the phosphatase activity of the receptor, or if further components of the PTPζ complex are needed to initiate dephosphorylation (51). Not only in the periphery, but also within the CNS, PTPζ has been shown to be involved in several MDK-dependent signaling pathways, including the promotion of neuronal survival (53) and the migration of neurons (45) (**Figure 1**), which is especially important during neurogenesis (54, 55).

### Anaplastic lymphoma kinase

The MDK/ALK signaling pathway is well established in diverse tissues and has been elucidated in numerous studies (43, 56–59). MDK binding to ALK results in phosphorylation of the insulin receptor substrate-1 (IRS-1), leading to enhanced activation of Src kinases (43), mitogen-activated protein (MAP)-kinase and phosphoinositide (PI)-3-kinase signaling (60), as well as the induction of the transcriptional activation of NF-κB (56) (**Figure 1**). As mentioned above, NF-κB acts as central mediator of inflammatory responses (61) and regulates fundamental cellular processes including differentiation, proliferation, and survival (57). In these lines, MDK/ALK signaling is especially involved in neoplastic diseases (18, 58, 62, 63), as it initiates, for example, an autocrine growth and survival signal via the suppression of caspases (58), as well as the enhancement of B-cell lymphoma-2 (Bcl-2) (62), an anti-apoptotic protein and oncogene. Both pathways counteract anti-tumor immunity (64, 65), thereby implicating MDK signaling in tumor resistance. In melanoma, MDK activates mTOR via a similar signaling pathway (**Figure 1**), leading to an increased expression of vascular endothelial growth factor receptor 3 (VEGFR3) and the stimulation of lymphangiogenic signals, resulting in metastatic growth in lymph nodes and the lungs (63).

In the CNS, MDK-dependent ALK signal transmission in glioma cells results in the activation of the Akt/mTOR1 axis (**Figure 1**), preventing autophagy-mediated cell death by tetrahydrocannabinol (THC), thereby contributing to the cannabinoid-resistance of gliomas (18). While MDK negatively contributes to cancer progression and metastasis formation via its anti-apoptotic and growth-promoting effects in the peripheral compartment as well as in the CNS, these functions may also have beneficial roles in the context of injuries and tissue regeneration. It is conceivable that the anti-apoptotic and proliferative effects of MDK possess the capacity to mediate tissue-protection and ameliorate inflammatory and demyelinating processes in the CNS (9, 66).

### Low-density lipoprotein receptor-related protein

In inflammatory diseases such as myocarditis, the interaction of MDK with the receptor LRP1 and members of the β_2_ integrin family is critical for MDK-induced PMN recruitment (**Figure 1**) and neutrophil extracellular trap (NET) formation (67). Here, MDK contributes to a process called NETosis (68), which results in inflammation and tissue injury through direct damage (67). In squamous cell carcinoma, MDK triggers phosphorylation and thereby activation of paxillin and signal transducer and activator of transcription (STAT) 1α pathways in an integrin-dependent manner, resulting in the overexpression of genes implicated in cell migration and tissue invasion (59).

In the CNS, binding of MDK to LRP1 has been shown to induce cell survival in embryonic neurons (44) (**Figure 1**), while MDK-signaling through the integrins α_4_ and α_6_ promotes neurite outgrowth (69). Upon binding of MDK to the receptor component LRP, MDK is internalized and transported to the nucleus (70). The nuclear translocation of the growth factor is enabled by the shuttle proteins nucleolin (71) and laminin binding protein precursor (LBP) (72), and necessary for the promotion of cell survival via the MDK/LRP signaling pathway (70) (**Figure 1**). Similar to the anti-apoptotic effects of MDK/ALK signaling in the CNS, MDK binding to LRP1 may thereby support regeneration and re-myelination in response to CNS insult.

### Neuroglycan C

In the CNS, the receptor neuroglycan C has been identified as important MDK signal transducer involved in process elongation of OPCs (49). These cells are not only important during synapse formation, but also for the re-myelination of axons in demyelinating diseases such as MS (73). Emerging literature on OPCs furthermore describes their potential for the establishment and remodeling of neural circuits (74), which supports the function of MDK in the developing brain. The receptor neuroglycan C might function in complex with LRP1 and integrins, which strongly bind to one another and may form the core of the MDK receptor complex in the CNS (69) (**Figure 1**).

In summary, the complex interplay of MDK receptor signaling facilitates the intricate and context-dependent functions of the growth factor in a broad variety of cell types, which regulate numerous inflammatory and non-inflammatory functions (**Figure 1**). Both, in the periphery and the CNS, MDK elicits pro-inflammatory and anti-apoptotic effects, driving inflammation, tumor progression, and metastasis. Nevertheless, upon injury or trauma, increased MDK expression has the potential to positively influence disease outcomes by promoting differentiation, reducing cell death, and increasing regeneration.

## MDK and its roles in injury, cancer, inflammation, and autoimmunity

**Figure 2:**
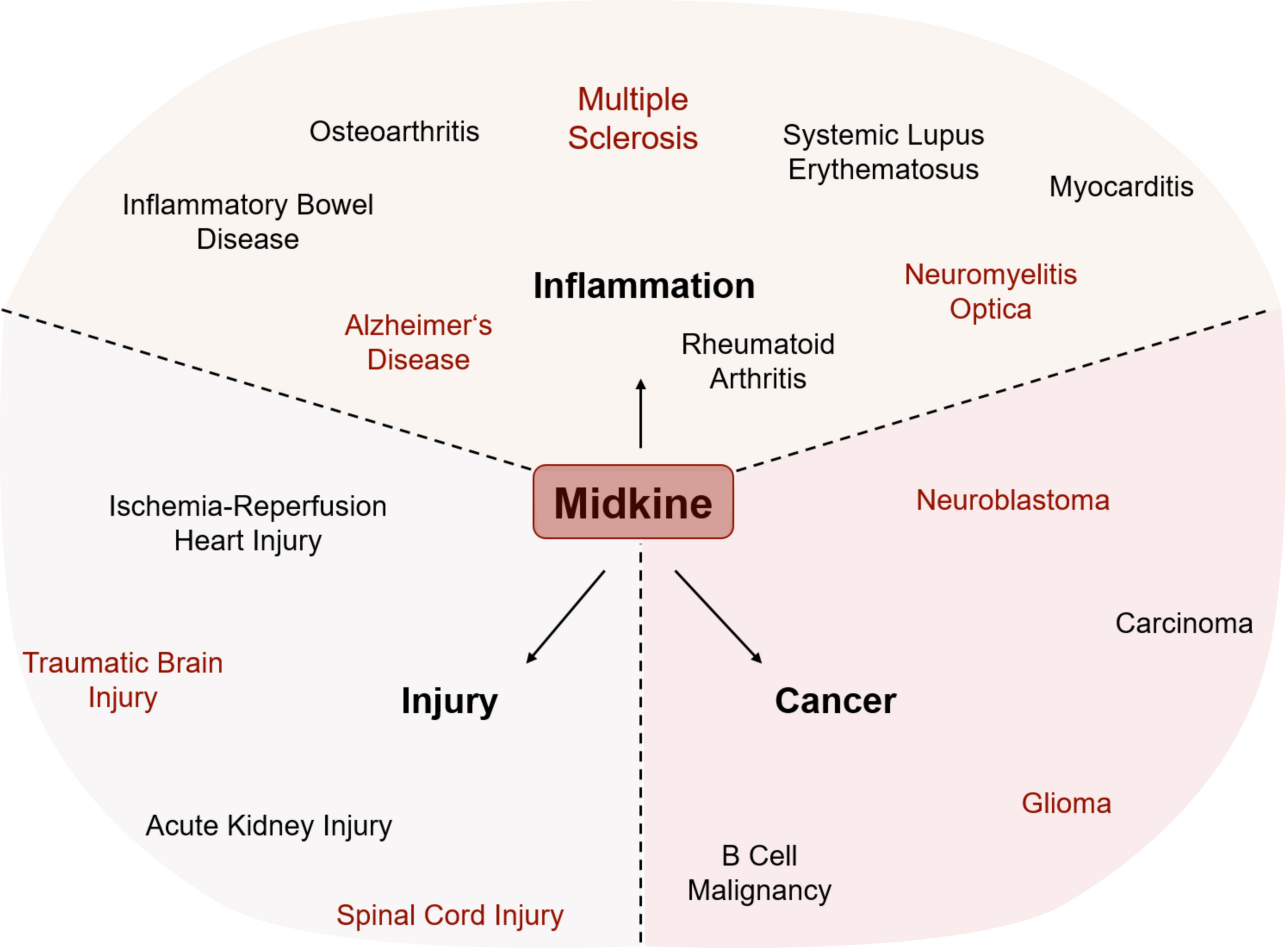
Involvement of MDK in various pathological conditions. The expression of Midkine (MDK) is increased in all shown conditions, while it has opposing functions within injury, inflammation, and cancer. MDK influences the outcome by either promoting recovery or deteriorating the course of disease and/or pathogenesis. Conditions written in red are central nervous system (CNS)-related injuries, brain tumors, or neuroinflammatory diseases.

Besides its important role during development and differentiation of various cell types (2), MDK has been shown to be upregulated in various pathological conditions in the periphery and the CNS, reaching from neoplastic diseases to inflammatory diseases and injuries (**Figure 2**, **Table 1**).

### MDK in the context of neoplastic diseases

In the context of neoplastic diseases, increased MDK levels have been demonstrated more than 20 years ago by several studies covering various types of cancer (**Figure 2**). In human tissue samples of prostatic (75) and hepatocellular (76) carcinomas, increased protein levels of MDK have been detected via immunohistochemical staining, while mRNA levels of MDK were increased in human gastric carcinoma specimens (77). *In vitro* studies suggest that MDK promotes proliferation and migration of pancreatic cancer cells (15) and is furthermore contributing to chemoresistance in ductal adenocarcinomas (16). These observations are supported by more recent studies in the context of breast cancer and melanoma, where MDK shapes the tumor microenvironment and promotes tumor-resistance (21, 23).

Aside from its numerous roles in tumor-formation and -resistance in peripheral tissues, MDK has also been implicated to play a role in the development and tumorigenicity of brain tumors (**Figure 2**). Among these, neuroblastomas belong to the most prevalent malignant pediatric solid tumors (78), while gliomas are the most frequent primary tumors of the CNS in adults (79).

*In vitro* studies using primary neuroblastomas and neuroblastoma cell lines suggest that MDK not only promotes peripheral neoplasms but is also involved in tumor growth and differentiation in the CNS (80). This hypothesis has been supported by reduced tumor growth in several MDK-depleted neuroblastoma cell lines (26). Moreover, elevated MDK blood levels can be linked to poor prognostic factors in neuroblastoma patients (81, 82), supporting the relevance of MDK in neuroblastoma tumorigenesis. Similarly, increased MDK levels in the CNS correlate with a poor prognosis and lower survival of glioblastoma patients (83), indicating an involvement of the growth factor in disease progression. A hallmark of glioblastomas is their ability to relapse in patients within a certain cell population, called glioma initiating cells (GICs), which exhibit stem-like characteristics (19). Because MDK has been implicated to promote the growth of neural stem cells and progenitor cells *in vitro* (84), it is likely that MDK is also involved in glioblastoma initiation. Studies with GIC cultures show increased MDK mRNA and protein levels, while inhibition of MDK reduces the ability of neurosphere generation by GICs, as well as the number of stemness biomarkers in culture (19).

Overall, these and other observations (16, 17) underscore the role of MDK signaling for chemoresistance and tumorigenesis in the context of solid brain tumors. These findings not only emphasize the relevance of MDK as a therapeutic target, but also illustrate its potential as early diagnostic and independent prognostic marker.

Although the functions of MDK in the context of solid tumors inside and outside the CNS exhibit remarkable similarities, it still remains unclear if the tumor promoting effects of MDK on peripheral and central neoplasms underlie a common mechanism. One conceivable common mechanism is mediated through the proto-oncogene p53. *MDK* is known to harbor p53 binding sites, where binding of the appropriate protein activates the transcription of MDK in gliomas, while knockdown of p53 downregulates the expression of mRNA and protein levels of MDK (20). In these lines, it has been suggested that the p53-induced overexpression of MDK in gliomas drives the anti-inflammatory polarization of microglia, thereby remodeling the tumor immunosuppressive microenvironment (20). Studies of low-grade gliomas (LGGs) using neurofibromatosis type 1 (NF1) as a genetic model system describe MDK as an upstream mediator regulating the activation of T cells, the release of cytokines, and thereby tumor growth in *NF1*-mutant murine and human neurons (85). Further reports describe the tumorigenic role of MDK in the context of NF1 (86, 87), indicating that MDK activation of T cells is a crucial mechanism in NF1-LGG pathogenesis. Additional commonalities include the control of MDK expression by NF-κB signaling and hypoxia. Activation of both pathways is a defining feature of the tumor microenvironment, irrespective of the tissue and cancer type (88). It is therefore conceivable that MDK is part of a common response mechanism to malignant tumors, and therefore potentially represents a central target for therapeutic intervention.

Overall, these findings demonstrate the importance of MDK as a central mediator of tumorigenesis, irrespective of tissue and cell type. Uncovering the exact signals that drive MDK expression and its transduction through its various binding partners in the tumor microenvironment is therefore of highest interest to identify novel therapeutic strategies that overcome tumor resistance.

### MDK in the context of autoimmune and inflammatory diseases

Besides its various functions in cancer, MDK has been described as an important regulator of autoimmune and inflammatory diseases (**Figure 2**). One of them is rheumatoid arthritis (RA), the most common inflammatory arthritis affecting joints as well as potentially other organs. The disease is characterized by synovial inflammation, hyperplasia, and the production of autoantibodies followed by cartilage and bone destruction (89). Main drivers of synovitis are leukocyte accumulation and the production of pro-inflammatory cytokines such as TNFα and IL-6 (89). MDK has been detected in inflamed synovial tissue of RA patients but not in healthy controls (90). Here, MDK leads to the activation and migration of neutrophils into inflamed tissue by either acting as chemoattractant or by inducing the release of pro-inflammatory cytokines including IL-8, IL-6, and CCL2 (30). Notably, the migration of inflammatory leukocytes into RA synovial tissue is suppressed in MDK knock-out mice (31), where disease activity is diminished. Similar observations have been made in the context of the autoimmune disease systemic lupus erythematosus (SLE). Its pathogenesis is characterized by the production of autoantibodies against nuclear and cytoplasmic antigens affecting several organs. Patients undergo periods of remission and relapse showing organ-specific symptoms (91). In SLE patients, elevated MDK plasma levels correlate with rash and increased levels of IL-17, a pro-inflammatory cytokine produced by T_H_17 cells (32). Increased levels of circulating MDK have not only been described in peripheral autoimmune diseases but also in other inflammatory conditions like ulcerative colitis (UC) (92) and Crohn’s disease (CD) (93), two main forms of inflammatory bowel diseases (IBDs) (**Figure 2**).

Additionally, MDK has been implicated in the regulation of primary degenerative and inflammatory diseases of the CNS (28, 94) (**Figure 2**). Alzheimer’s disease (AD), for instance, is a complex neurodegenerative disorder and one of the major causative factors for cognitive impairment. Molecular hallmarks of its pathogenesis include plaque formation by extracellular aggregates of β-amyloid (Aβ) peptides and intracellular neurofibrillary tangles made of hyperphosphorylated tau (τ) protein (95). While AD is not considered a primary inflammatory disorder, it has become increasingly clear that secondary inflammation is a key driver of disease progression (96, 97). In these lines, increased MDK levels have been found in serum and plaques of AD patients (94). These observations match the increase in inflammatory markers in AD patients and support the idea of a close interaction between amyloid pathology and inflammation. In this context, MDK has been shown to inhibit Aβ fibril formation and Aβ-induced cytotoxicity (11, 12), highlighting the tissue-protective potential of the growth factor in AD.

**Figure 3:**
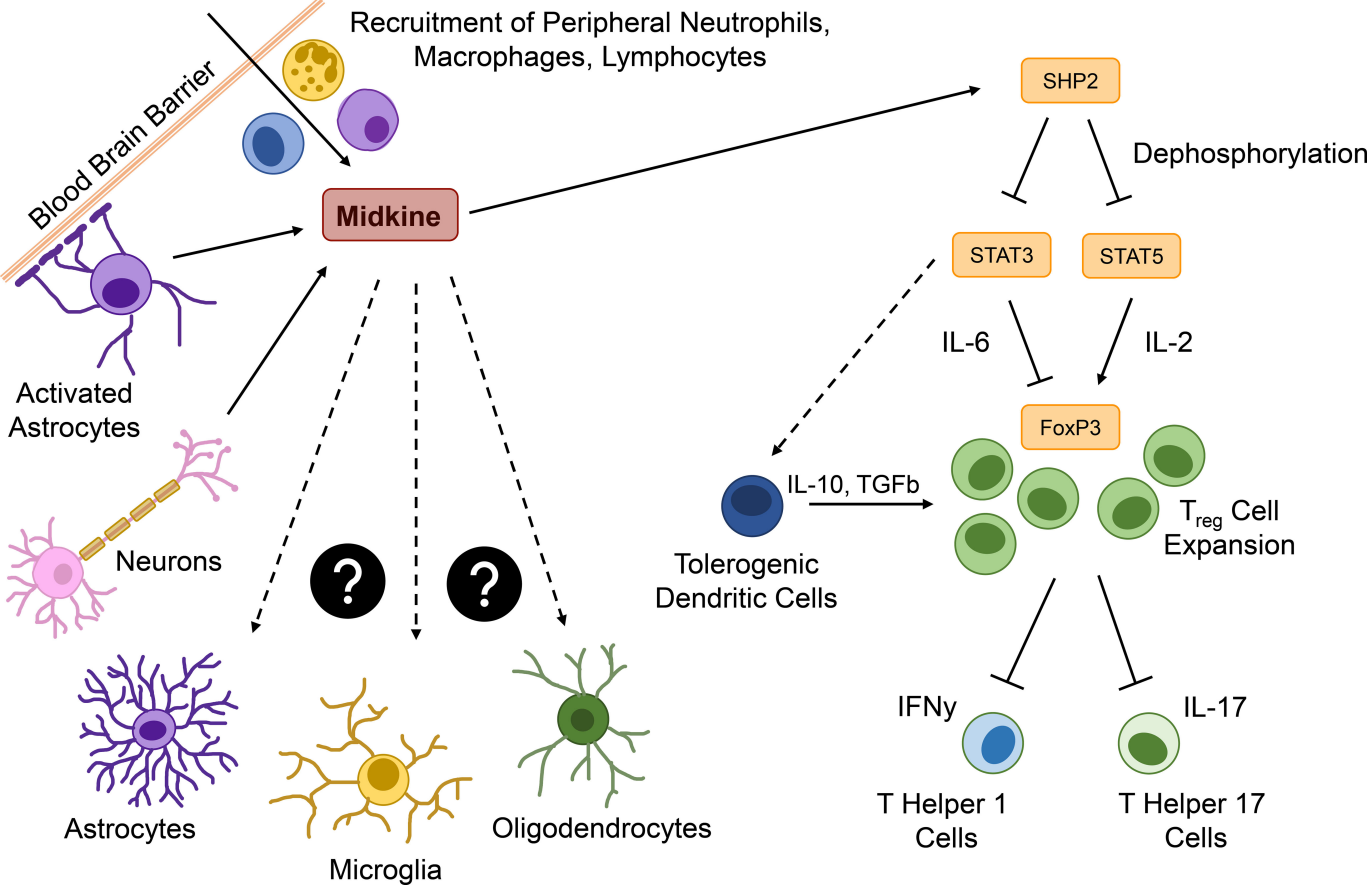
Major functions of MDK in the context neuroinflammation. Within the brain, Midkine (MDK) is expressed by activated astrocytes or neurons and acts as a chemoattractant for peripheral immune cells, such as neutrophils, macrophages, and lymphocytes. MDK promotes the expression of the tyrosine phosphatase SHP2, which dephosphorylates signal transducer and activator of transcription (STAT) 3 and 5. STAT5 usually induces the expression of the transcription factor forkhead-box-protein p3 (FOXP3) in an interleukin (IL)-2-dependent way, promoting the expansion of regulatory T (T_reg_) cells. STAT3 is required for the IL-6-dependend inhibition of Foxp3 expression and might be involved in the induction of tolerogenic dendritic cells (DC_reg_), which additionally promote a T_reg_ cell expansion. T_reg_ cells downregulate the interferon γ (IFNγ) expressing T helper (T_H_) 1 and the IL-17 expressing T_H_17 cells. The pro-inflammatory cytokine IL-23 triggers pathological features in IL-17 producing T cells and might be in correlation with MDK expression. All MDK-induced events lead to continuous neuroinflammation, while effects of MDK on central nervous system (CNS) resident cells such as astrocytes, microglia, and oligodendrocytes is still unknown.

In autoimmune CNS disorders like neuromyelitis optica (NMO) and MS, increased levels of MDK have been associated to a poor prognosis (28). This is in line with reports of a direct correlation between MDK serum levels and IL-23 levels (98), a pro-inflammatory cytokine that drives pathological functions of T_H_17 cells (99). Moreover, MDK mRNA expression in mice is highly upregulated upon EAE induction and correlates with disease progression and clinical symptoms (100). *In vivo* studies using MDK-deficient mice describe an expansion of regulatory T (T_reg_) cell populations upon EAE induction, which in turn reduces the numbers of autoreactive T_H_1 and T_H_17 cells (**Figure 3**), resulting in disease amelioration compared to control mice (25). T_reg_ cell development is regulated through the transcription factors STAT3 (24) and STAT5 (25) and based on a MDK-dependent suppression of tolerogenic dendritic (DC_reg_) cells, which usually promote T_reg_ cell differentiation (24) (**Figure 3**). The functional relevance of MDK in the context of EAE is further supported by observations of decreased inflammatory infiltration in spinal cords of MDK-deficient mice, concomitant with reduced disease severity compared to controls (25). The beneficial outcomes of MDK deficiency in EAE can be reversed by exogenous application of recombinant MDK, which exacerbates disease severity and indicates an overall detrimental function of the growth factor in autoimmune neuroinflammation (25).

In conclusion, MDK plays important roles in the onset and progression of autoimmune and inflammatory diseases in the periphery and the CNS. MDK serum levels are elevated in patients with inflammatory, autoimmune, and neurodegenerative diseases, while *in vivo* studies reveal that MDK contributes to inflammation via the induction of pro-inflammatory cytokines, the recruitment and activation of inflammatory immune cells, as well as the suppression of regulatory mechanisms (**Figure 3**). While these data support the notion that MDK is an important mediator of inflammation not only in peripheral pathologies but also following CNS insult, future studies are needed to delineate mechanisms and target cells in the CNS (**Figure 3**).

### MDK in the context of acute injury

Aside from neoplastic diseases and primary inflammatory disorders, upregulation of MDK can also be observed in injuries of peripheral organs such as the heart or the kidney (66, 101) (**Figure 2**). Upon renal ischemia-reperfusion, a process frequently leading to excessive tissue injury and destructive inflammatory responses (102), MDK promotes the migration of neutrophils and macrophages to the site of injury (29) while in cardiac ischemia-reperfusion injury MDK prevents myocardial apoptosis (13).

Similarly, in the injured CNS, numerous functions of MDK have been proposed. Traumatic brain injury (TBI) starts with primary tissue damage directly caused by the insult, followed by secondary tissue damage, which is induced by pathological processes after the primary insult and leads to necrosis and apoptosis of cells in the CNS (103). Major consequences of traumatic insults are blood brain barrier (BBB) breakdown, subsequent infiltration of immune cells into the brain (104), and neuroinflammation. *In vivo* studies with wild type and MDK-deficient mice demonstrated that MDK-deficiency does not affect astrogliosis following TBI (34), confirming earlier results of *in vitro* experiments, where MDK treatment of purified astrocyte and microglia cell cultures did neither induce astrogliosis nor microgliosis (37). Astrogliosis is a process in which astrocytes respond to CNS damage or disease by transcriptional remodeling and an altered activation state (105). Depending on the severity and permanence of this state, astrogliosis is associated to beneficial and necessary functions, but can also lead to harmful effects by cellular hypertrophy, proliferation, and the secretion of pro-inflammatory cytokines (106, 107). The same is true for microglia, the tissue-specific macrophages of the CNS (108). Following their activation, microglia can exert neurotrophic as well as neurotoxic functions (108). However, while treatment of microglia with MDK *in vitro* resulted in no major alterations, Takada et al. (34) observed a shift to an anti-inflammatory microglia polarization state during the acute phase of TBI in MDK-deficient mice. In addition, the authors observed that MDK-deficiency leads to a decrease in apoptotic neurons around lesions, thereby reducing cerebral atrophy and neurological deficits after TBI (34). As the growth factor also features chemoattractant properties, especially the recruitment of neutrophils and macrophages (34, 109) (**Figure 3**), it is conceivable that increased BBB permeability upon primary traumatic insult allows MDK to amplify the recruitment of peripheral immune cells and thereby potentiates secondary injury (34). Indeed, a reduction in the transgression of immune cells into the CNS was observed in MDK-deficient mice, supporting the notion that MDK regulates immune cell infiltration in the context of TBI.

Aside from TBI, MDK is expressed in early stages of cerebral infarct, a condition where the blood supply to the brain is disrupted, leading to ischemia and hypoxia, and finally to necrotic tissue in the brain. MDK has been detected at the sites of nerve damage, where it seems to act as a reparative neurotrophic factor (14). These findings align with the transcriptional regulation of MDK by HIF-1α (**Figure 1**) and highlight the reparative potential of MDK in hypoxia-driven disorders. *In vivo* studies in rat showed an upregulation of MDK mRNA, as well as protein levels following transient forebrain injury (33) and increased expression of MDK in damaged areas of traumatic spinal cord injury in regards to tissue repair (110).

These data collectively suggest that MDK is part of a central inflammatory response mechanism that governs injury responses, as well as numerous autoimmune-, inflammatory-, and cancer pathologies (**Figure 2**, **Table 1**). Depending on the inflammatory state and the microenvironment at the site of injury MDK exerts opposing functions and either promotes the amplification or suppression of pathological processes. Due to increased MDK levels in several diseases, the growth factor may be of high relevance as disease marker and target for drug development. Especially in the CNS, where MDK may drive the infiltration of peripheral immune cells and the pro-inflammatory activation of glial cells during acute insult, its protective functions on microglia, oligodendrocytes, and neurons underscore its therapeutic potential for regenerative processes in response to acute CNS insult. However, further studies are needed to clarify MDK signaling pathways involved in CNS pathologies, but also cancer progression, metastasis, inflammation, and other peripheral pathological conditions.

## MDK as a mediator of neuro-immune crosstalk

The regulatory functions of MDK are indispensable during development, where the growth factor mediates embryogenesis, organogenesis, as well as neurogenesis (111). While in healthy adults, MDK is only expressed in the kidney, several pathological conditions are accompanied by an increase in MDK levels in the periphery, as well as the CNS (10, 36) (**Figure 2**). In these lines, it is becoming increasing clear that MDK is not only an important mediator of disease processes within a specific compartment, but also functions as mediator of neuro-immune cell-to-cell crosstalk.

While under homeostatic conditions, the CNS is shielded from the periphery by the BBB, inflammation induced barrier dysfunction may foster the MDK-dependent interaction between CNS-resident and peripheral cell types. In these lines, the infiltration of MDK-expressing immune cells through a leaky BBB may stimulate context-specific MDK-signaling events in CNS-resident cells, or *vice versa* (**Figure 3**). While the effects of MDK on glial cells are not fully understood, MDK has been shown to induce an anti-inflammatory polarization state in microglia *in vivo* (34). Even though there are no direct effects on astrocytes revealed so far (34), MDK-induced polarization of microglia might regulate crosstalk between glial cells (112) and thereby indirectly modulate the functions of astrocytes, as well as other CNS-resident cells, such as neurons and oligodendrocytes, which may exert the described neuroprotective effects of MDK (84, 113). On the other hand, MDK expressed by CNS-resident cells upon insult or inflammation may act as a mediator of neuro-immune crosstalk by promoting the recruitment of peripheral immune cells through a leaky BBB into the CNS (**Figure 3**), thereby fueling inflammatory processes within the CNS and ultimately leading to disease deterioration and additional activation of glial cells. The importance of MDK as a mediator of neuro-immune crosstalk is furthermore exemplified by its role in the suppression of regulatory functions within the CNS. Here, the secretion of MDK by CNS-resident cells induces tyrosine phosphatase SHP2 expression, which dephosphorylates and thereby inactivates STAT3 and STAT5. This cascade results in the suppression of DC_reg_ cells, and consequently T_reg_ cells, leading to increased numbers of effector T cells (**Figure 3**), and the exacerbation of inflammatory processes *in vivo* (24, 25).

Similar mechanisms of MDK may contribute to the development and pathogenesis of neoplastic diseases, where neurons, microglia, macrophages, and T cells in the tumor microenvironment control formation, growth, and progression of malignant solid tumors (85, 114–118). Here, MDK not only recruits peripheral immune cells, but also activates CD8^+^ T cells, establishing a neuro-immune-cancer axis that promotes tumor growth (85). The exact routes and mechanisms of crosstalk, and how MDK derived from the periphery versus CNS-derived MDK regulates inflammatory reactions still need to be addressed in future studies.

## MDK as putative biomarker

Due to the distinct expression of MDK in various pathological conditions, especially malignancies and inflammatory diseases (**Figure 2**, **Table 1**) the growth factor has been considered as a putative biomarker (36, 119–121). While the potential of MDK as a biomarker has been proposed for several cancer types and inflammatory diseases, further studies are required to delineate its specificity as a biomarker. In hepatocellular carcinoma (HCC), MDK enables a discrete discrimination of patients with early HCC from those with cirrhosis (122). The assessment of serum and urinary MDK levels furthermore facilitates the early detection of non-small cell lung cancer (NSCLC) (123) and aids clinical decision making, as high MDK levels correlate with poor prognosis in NSCLC patients (124). Recently, the growth factor MDK has additionally been described as candidate biomarker in lung adenocarcinoma, one of the most common types of lung cancer (125). As MDK is a systemic lymphangiogenesis-inducing factor, its detection might function as a prognostic marker for melanoma patients (63). In brain tumors both MDK and PTN might be useful as early diagnostic and independent prognostic markers, as MDK overexpression correlates with the rapid progression of astrocytomas (126) and a poor survival outcome in high-grade gliomas (127). Studies in the context of autoinflammatory diseases such as RA, SLE, UC, and CD have proposed MDK as a marker for the detection of inflammatory disease activity (92, 93, 128, 129), with a performance comparable to, and potentially superior to established disease activity markers like C-reactive protein (CRP) (92, 93). Finally, a recent has described MDK levels in the cerebrospinal fluid of Parkinson’s disease (PD) patients as a supportive diagnostic biomarker (130), highlighting its potential for other neurodegenerative disease such as MS or AD.

## MDK as therapeutic target

Beyond its significance as a biomarker, the involvement of MDK in numerous diseases, including injuries, malignancies, and inflammatory disorders of the periphery and CNS (**Table 1**), harbors significant potential as a therapeutic target. Depending on the type of disease and the function of the growth factor within a pathological condition, therapeutic approaches could consist of MDK blockage or the exogenous supplementation of MDK.

In neoplastic diseases, MDK promotes tumor growth, differentiation, and therapy-resistance. In these lines, MDK-targeted strategies may have great therapeutic potential, particularly in refractory cancer settings. Recent studies demonstrate that blockage of MDK signaling by various approaches rescues tumor resistance. For instance, the use of the small molecule inhibitor iMDK (131), small interfering RNAs (siRNAs) (132), or MDK blockage using anti-MDK monoclonal antibodies (133) restores tumor apoptosis and inhibits tumor growth in mice. A promising human MDK blockade system has already been established *in vitro* using prostate cancer xenografts, where synthetic siRNA in combination with the chemotherapeutic paclitaxel (PTX) affects tumor cell proliferation, apoptosis, and angiogenesis (132). Another way to target MDK is via an antisense oligodeoxynucleotide molecule based on the secondary structure of MDK mRNA, referred to as antisense oligoDNA, or morpholino antisense oligomers. Treatment with antisense MDK suppresses tumorigenicity in mouse rectal carcinoma cells and other xenograft models *in vitro* and reduces tumor growth in nude mice *in vivo* (134). A recent study precisely looked into the effects of MDK within the HCC microenvironment and postulated MDK inhibition as valuable therapeutic addition to anti-PD-1 immunotherapy in HCC patients, as the standard treatment, sorafenib, leads to an immunosuppressive tumor microenvironment due to increased MDK expression (135). MDK-TRAP, a MDK-binding peptide derived from the MDK receptor LRP1, inhibits, similar to anti-MDK antibodies, the binding between MDK and LRP1, thereby decreasing cell growth and colony formation in G401 cells and CMT-93 cells (136). As MDK/LRP1 signaling contributes to anchorage-independent tumor cell growth, its disruption might be a promising cancer treatment approach, along with MDK-TRAP and polyclonal antibodies. The blockage or inhibition of MDK-mediated effects prior to or during chemotherapy might increase treatment effectiveness and benefits patients who are not responding to conventional treatments.

Therapies with siRNA, oligoDNA, and other drugs inhibiting MDK have not yet been tested for CNS-related neoplastic diseases but might represent enormous therapeutic potential for the treatment of glioblastomas. While in neuroblastomas PTN expression is linked to good prognosis, high MDK mRNA levels are detected in tumors with poor prognosis (80). This is of particular interest, as knockout of PTN and its receptor ALK exerts antitumorigenic effects in glioblastoma animal models (137). Monoclonal antibodies directed against MDK may allow targeting these tumorigenic effects in the CNS, however, current candidates still lack the necessary efficacy (138, 139). Moreover, RNA aptamers against MDK hold great potential for therapeutic treatment of neuroblastomas (27). Aptamers are biochemical agents that specifically recognize a particular target, usually a protein (140). They bind their target with high affinity and function similarly to antibodies, which is why they have been considered as highly effective therapeutics. *In vitro* and *in vivo* studies with tumor xenografts depict a suppressed growth of neuroblastoma cells upon intratumoral administration of RNA aptamers specific for MDK (27). The clinical efficacy of anti-MDK aptamers has additionally been shown for autoimmune disorders of the CNS, such as MS. Anti-MDK aptamers induce T_reg_ cell expansion *in vitro*, while treatment of EAE mice with MDK-specific RNA aptamers results in a delayed disease onset and lower clinical scores (25). This attenuation of autoinflammatory processes has also been observed when anti-MDK RNA aptamers were administered post EAE onset, once the disease is established, demonstrating its therapeutic potential in a clinically relevant setting (25).

Collectively, the blockade of MDK harbors great potential as therapeutic strategy in neoplastic and autoimmune diseases of both the periphery and the CNS. Nonetheless, it is important to better understand the upstream and downstream regulators of MDK signaling in order to develop novel therapeutic strategies.

In contrast to MDK-targeting strategies that aim to reduce MDK levels in target tissues, several approaches have been proposed that incorporate distinct features of the growth factor or focus on an exogenous or endogenous increase of MDK levels. In these lines, MDK might be a candidate for cancer vaccine development, as it has been shown that MDK-primed cytotoxic T cells are able to lyse tumor cells (36). Another novel therapeutic strategy for peripheral tumors expressing MDK is the promotor-based conditionally replicative adenovirus therapy, which has been tested in pancreatic cancer cell lines *in vitro* (141). This gene therapy involves an oncolytic virus containing part of the *MDK* promotor, named Ad-MDK. The virus is capable of killing tumor cells and even though the growth factor MDK itself is not involved in this kind of therapy, its solely expression in cancer tissues allows a tumor-selective replication of the virus containing the *MDK* promotor and might be a promising new cancer therapy (141). As shown for pancreatic carcinomas, Ad-MDK gene therapy enables glioblastoma-specific expression of oncolytic viruses, highlighting the use of MDK for the treatment of malignant glioblastomas (142). Gene therapy might also be a useful tool in non-neoplastic CNS-affecting diseases as MDK is thought to be involved in neural repair upon brain injuries. Studies in mice show that the injection *MDK* encoding adenovirus after ischemic injury decreases the infarct volume and protects against ischemic damage (143, 144). Similarly, intrathecal administration of MDK promotes functional recovery upon spinal cord injury in rats (37), supporting the beneficial effects of elevated MDK levels following CNS insult (113). Altogether, the endogenous or exogenous elevation of MDK levels in the CNS represents a promising treatment option for various injuries of the nervous system. So far, multiple non-invasive approaches for drug delivery into the CNS have been tested, including intranasal administration (145–147), focused ultrasound (148) or nanobiotechnology-based delivery techniques (149). These approaches may harbor great potential for the exogenous elevation or reduction of MDK levels in the CNS. Additionally, gene and cellular therapies may represent useful long-term strategies for numerous CNS-affecting disorders (150).

## Conclusion

The multifunctional growth factor MDK is a central factor in numerous pathologies (**Table 1**, **Figure 2**) and harbors great potential as biomarker and therapeutic target (121). Depending on disease, MDK exerts diverse functions that drive or suppress disease progression. As we have discussed in this review, MDK exerts tumorigenic functions by promoting tumor growth, differentiation, and chemoresistance in neoplastic diseases (16, 26, 27, 78, 82). Additionally, MDK contributes to the onset and progression of inflammatory and autoimmune diseases through its chemoattractant properties (29–31, 90) and the suppression of regulatory mechanisms (24, 25). While these mechanisms collectively contribute to disease progression, it has become clear that MDK can also exert tissue-protective functions (11, 12) that attenuate neurodegeneration and support repair in the periphery (13, 29) and CNS (34).

Altogether, these diverse functions allow a wide range of MDK-centered therapeutic strategies. Numerous studies have already demonstrated beneficial outcomes following MDK blockade in inflammatory disorders and malignancies (25, 131–134).

The next step is now to evaluate these strategies in combination with established therapies in order to increase treatment efficacy and to overcome tumor-resistance. Moreover, as central mediator of neuro-immune crosstalk, MDK has great potential as therapeutic target in CNS disorders. While inhibition or blockade of MDK signaling may be a promising option for neoplastic, inflammatory, or autoimmune diseases affecting the CNS, endogenous or exogenous increase of MDK levels could improve the outcome in the context of acute CNS injuries and ischemia. In these lines, particularly recently emerging opportunities of non-invasive drug delivery into the CNS further support the therapeutic potential of MDK-centered therapies in the treatment of CNS disorders (145–149). Finally, beyond its functions as therapeutic target and a critical modulator of disease processes, MDK offers great potential as putative biomarker in the context of various malignancies and disorders (92, 93, 122–124, 127–129). Future studies will be necessary to evaluate each individual benefit of MDK as a biomarker and compare them to well established markers. In summary, MDK unveils new therapeutic avenues that necessitate further validation in future studies.

## Author contributions

EN: Writing – original draft, Writing – review & editing. VR: Writing – review & editing. ML: Writing – original draft, Writing – review & editing.
